# Performance of spleen stiffness measurement by 2D-shear wave elastography in evaluating the presence of high-risk varices: comparative analysis of idiopathic portal hypertension versus hepatitis B virus

**DOI:** 10.1186/s12880-023-00977-9

**Published:** 2023-02-09

**Authors:** Huihui Zhou, Zhilin Zhang, Jun Zhang, Lin Sang, Lina Liu, Xue Gong, Yuanyuan Sun, Yu Zheng, Ming Yu

**Affiliations:** 1 Department of Ultrasonic Medicine, The First Affiliated Hospital of Fourth Military Medical University, Xi’an, 710032 Shaanxi China; 2 Department of Ultrasound, Xi’an Central Hospital, Xi’an, 710003 Shaanxi China

**Keywords:** Two-dimensional shear-wave elastography, Liver stiffness, Spleen stiffness, High-risk varices, Idiopathic portal hypertension, Hepatitis B virus

## Abstract

**Background:**

Noninvasive assessment of high-risk varices (HRV) in idiopathic portal hypertension (IPH) is rare. The purpose of this study was to investigate the performance of spleen stiffness (SS) for evaluating the presence of HRV in IPH patients as compared the measurements in patients with hepatitis B virus (HBV).

**Methods:**

A retrospective single-center study was performed to evaluate the performance of SS for assessing HRV in IPH and HBV-infected patients, in comparison with liver stiffness (LS), spleen stiffness-to-liver stiffness ratio (SS/LS), LS spleen-diameter-to-platelet-ratio score (LSPS), portal hypertension risk score (PH risk score) and varices risk score, by using upper gastrointestinal endoscopy (UGE) as the gold standard. Finally, 86 IPH and 102 HBV-infected patients were enrolled. UGE, two-dimensional shear wave elastography (2D-SWE) and laboratory data were collected, and noninvasive parameters were calculated. Analysis of receiver operating characteristic (ROC) curves was conducted to acquire the optimal area under the ROC curve (AUC) and cutoff value for predicting the presence of HRV.

**Results:**

In patients with HRV, the significantly different parameters between IPH (34.9%) and HBV-infected patients (46.1%) were as follows: spleen size (diameter 18.5 ± 3.9 cm vs. 20.8 ± 2.7 cm), SS (50.2 kPa vs. 42.9 kPa), LS (11.1 kPa vs. 18.3 kPa) and PT (prothrombin time 15.1 s vs. 16.7 s). No statistically significant differences were found in liver function, platelet counts, spleen thickness and flow volumes in the portal venous system (*p* > 0.05). The AUCs of SS were 0.98 and 0.96 for predicting the presence of HRV in IPH (44.0 kPa cutoff value; 0.93 sensitivity; 0.96 specificity) and HBV-infected patients (35.2 kPa cutoff value; 1.00 sensitivity; 0.82 specificity), respectively, which were significantly better than other parameters.

**Conclusion:**

SS shows the optimal overall performance for predicting the presence of HRV in IPH and HBV-infected patients, in comparison with other noninvasive parameters.

## Background

Idiopathic portal hypertension (IPH) is a relatively rare disease characterized by portal hypertension (PH) in the absence of causative disease, such as cirrhosis, chronic liver disease and occlusion of the extrahepatic portal vein or hepatic vein [1]. The main clinical signs of this disease are PH and portal hypertension-related complications, including variceal bleeding, splenomegaly, hypersplenism, ascites and hepatic encephalopathy. These are also the main factors that affect the prognosis of patients [1]. Up to now, the pathophysiological mechanisms of IPH are poorly understood and the progression cannot be presented. Therefore, the important treatment for managing PH and its related complications as recommended in IPH [2]. Gastroesophageal varices (GEVs) are a progressive condition in IPH, and the accurate evaluation of the severity of GEVs is important for the prognosis, surveillance and management of IPH.

Upper gastrointestinal endoscopy (UGE) is considered the gold standard for predicting the severity of GEVs. However, it has several limitations: invasive, carry complications risk, costly and require a specific expertise [3]. Several noninvasive parameters based on noninvasive ultrasonic elastography technologies and/or laboratory markers, such as liver stiffness (LS), spleen stiffness (SS), spleen stiffness-to-liver stiffness ratio (SS/LS), LS spleen-diameter-to-platelet-ratio score (LSPS), portal hypertension risk score (PH risk score) and varices risk score, are used to predict the severity of GEVs in patients with chronic liver disease [4–6], but their diagnostic performance remains unknown in IPH. Very few studies, which also have small sample sizes, have been performed to evaluate the diagnostic performance of LS by using transient elastography (TE) in IPH patients [7]. In addition, the performance of SS measurement based on elastography technologies has been demonstrated in patients with chronic liver disease by many studies [8, 9].

Two-dimensional shear wave elastography (2D-SWE) was demonstrated to be a more effective noninvasive tool by several studies, as compared to TE, acoustic radiation force impulse imaging (ARFI), and point shear wave elastography (pSWE), and it could obtain a higher success rate in patients with obesity, ascites and narrow intercostal windows [9]. Meanwhile, it could acquire more accurate tissue stiffness values, because of combining B-mode imaging with a color-coded tissue stiffness map in real time, so that organ capsule, vessels and bile ducts can be effectively avoided. In addition, the cutoff values of different techniques have obvious specificity [10, 11].

Therefore, in our study, we aimed to clarify the diagnostic performance of SS by using 2D-SWE for predicting the presence of high-risk varices (HRV) in IPH patients compared with hepatitis B virus infected (HBV-infected) patients. Herein, the current retrospective single-center comparative study was designed.

## Methods

### Study design

This was a retrospective single-center comparative study that aimed to assess the performance of SS for predicting the presence of HRV in IPH patients and compared to the performance in HBV-infected patients. UGE was used as the gold standard, and SS was compared with LS, SS/LS, LSPS, PH risk score and varices risk score. Between December 2015 and December 2021, a total of 188 patients were enrolled, including patients with IPH (45.7%, 86 of 188) and HBV (54.3%, 102 of 188). The retrospective study was carried out in accordance with releveant guidelines and regulations or declaration of Helsinki and was approved by the ethics committee of the First Affiliated Hospital of Fourth Military Medical University. Informed consent was waived for the retrospective single-center comparative study. All authors accessed the study data and reviewed and approved the final manuscript.

### Patient population

The inclusion criteria were as follows: (1) age 18–75 years; (2) IPH diagnosed by liver biopsy; (3) HBV-infected patients with HBsAg positive more than 6 months and no other chronic liver disease; and (4) UGE, noninvasive examinations (2D-SWE examinations and abdominal Doppler US) and laboratory tests within 7 days. The exclusion criteria were as follows: (1) previous treatment with non-selective beta-blockers, shunt placement, surgical treatment, band ligation, liver transplantation, splenectomy and overt hepatic encephalopathy; (2) intrahepatic or extrahepatic malignancies; (3) portal vein thrombosis or cavernous transformation diagnosed by Doppler US or computed tomographic (CT); (4) companied with other chronic liver disease, including autoimmune hepatitis, any other viral hepatitis and alcoholic hepatitis; (5) 2D-SWE examination failed; (6) missing important laboratory data; and (7) female patients who were pregnant or lactating. Finally, the demographic and clinical information of the patients were recorded, including gender and age.

### Abdominal US and two-dimensional shear wave elastography examinations

Abdominal US and 2D-SWE examinations were conducted by using the Aixplorer system (SuperSonic Imagine; Aix-en-Provence, France) with a convex broadband transducer (SC6-1, frequency of 1–6 MHz). All ultrasound-related examinations were performed by two experienced sonographers who conducted at least 1000 abdominal US and 1000 2D-SWE examinations and were blinded to the clinical information and serological results.

All patients fasted for at least 4 h before the examinations. During the examinations, each patient was placed in the dorsal decubitus position with their arms maximally lifted, which allowed for full view of the epigastrium. Firstly, spleen size, the diameters and velocity of portal venous system, and heart rate were obtained by using conventional US, and then the flow volumes in portal venous system were calculated. Secondly, the LS (a 4 × 3 cm box) and SS (a 3 × 3 cm box) were measured by using 2D-SWE through the right and left intercostal windows, respectively. Patients needed to hold their breath (neither at full inspiration nor at full expiration at the end of expiration) for approximately 5 s, and effective 2D-SWE images were acquired, in which the region of interest (ROI) filled at least 85% of the color map and was stabilized for approximately 5 s. Then, the activated Q-box system (diameter range 5–20 mm) was placed in a parenchyma location, avoiding large vessels, biliary tracts and focal lesions, and its depths were 2 cm below the organ’s capsule. According to early reports [12], at least five 2D-SWE successful measurements were performed, and then median values were calculated in liver and spleen for each patient, respectively. Finally, the mean value of Young’s modulus was used for statistical analysis.

### Endoscopic assessment

All UGE examinations were performed by two endoscopists with more than 8 years of experience. The GEVs results were recorded as the LDRF classification described by Li et al. [13], which was used in the National Clinical Research Center for Digestive Diseases and First Affiliated Hospital of Fourth Military Medical University of Digestive Diseases (the high-level teaching hospital in China). The definition of HRV that was used in our hospital was previously described by Karagiannakis et al. and the definitions were as follows: esophageal varices sizes at least 5 mm, varices with red wales irrespective of size, and varices with any gastric varices [8].

### Serological data

All serological data (including liver function, blood counts and coagulation tests) were extracted from the institutional electronic medical records. On the basis of biochemical markers, the noninvasive scores were calculated as reported previously as follows: SS/LS = spleen stiffness value/liver stiffness value [14], LSPS = [LS (by using either TE or SWE and given in kilopascals) × spleen diameter (in centimeters)]/platelet count ratio (× 10^9^/L) [4], PH risk score = − 5.953 + 0.188 × LS (by using either TE or SWE and given in kilopascals) + 1.583 × sex (1: male; 0: female) + 26.705 × spleen diameter (in millimeters)/platelet count (× 10^9^/L) ratio [5, 6], varices risk score = − 4.364 + 0.538 × spleen diameter (in millimeters) − 0.049 × platelet count (× 10^9^/L) − 0.044 × LS + 0.001 × [LS × platelet count (× 10^9^/L)] [5].

### Statistical analysis

The continuous variables were expressed as the means ± standard deviations (SD) or medians [interquartile ranges (IQR)], depending on whether the variables followed a normal or non-normal distribution, whereas the categorical variables were expressed as numbers and percentages, when appropriate. For the analysis of the participants’ baseline characteristics, the continuous variables between groups were analyzed by Student’s t test or the Mann–Whitney U test, when appropriate. Categorical variables were compared by the Chi-square test or Fisher’s exact test, when appropriate. The diagnostic performance of noninvasive parameters for predicting the presence of HRV was estimated by receiver operating characteristic (ROC) curves. Differences between the areas under the ROC curves (AUCs) were compared by using the DeLong test. The sensitivity, specificity, positive predictive value (PPV), negative predictive value (NPV), positive diagnostic likelihood ratio (LR+) and negative diagnostic likelihood ratio (LR−) were calculated. All statistical analyses were two sided, and *p* values less than 0.05 indicated statistical significance.

Statistical analyses were performed using SPSS software (version 26; IBM, Armonk, NY, USA) and MedCalc software (V.11.2; 2011 MedCalc Software bvba, Mariakerke, Belgium).

## Results

### Patient characteristics

**Fig. 1:**
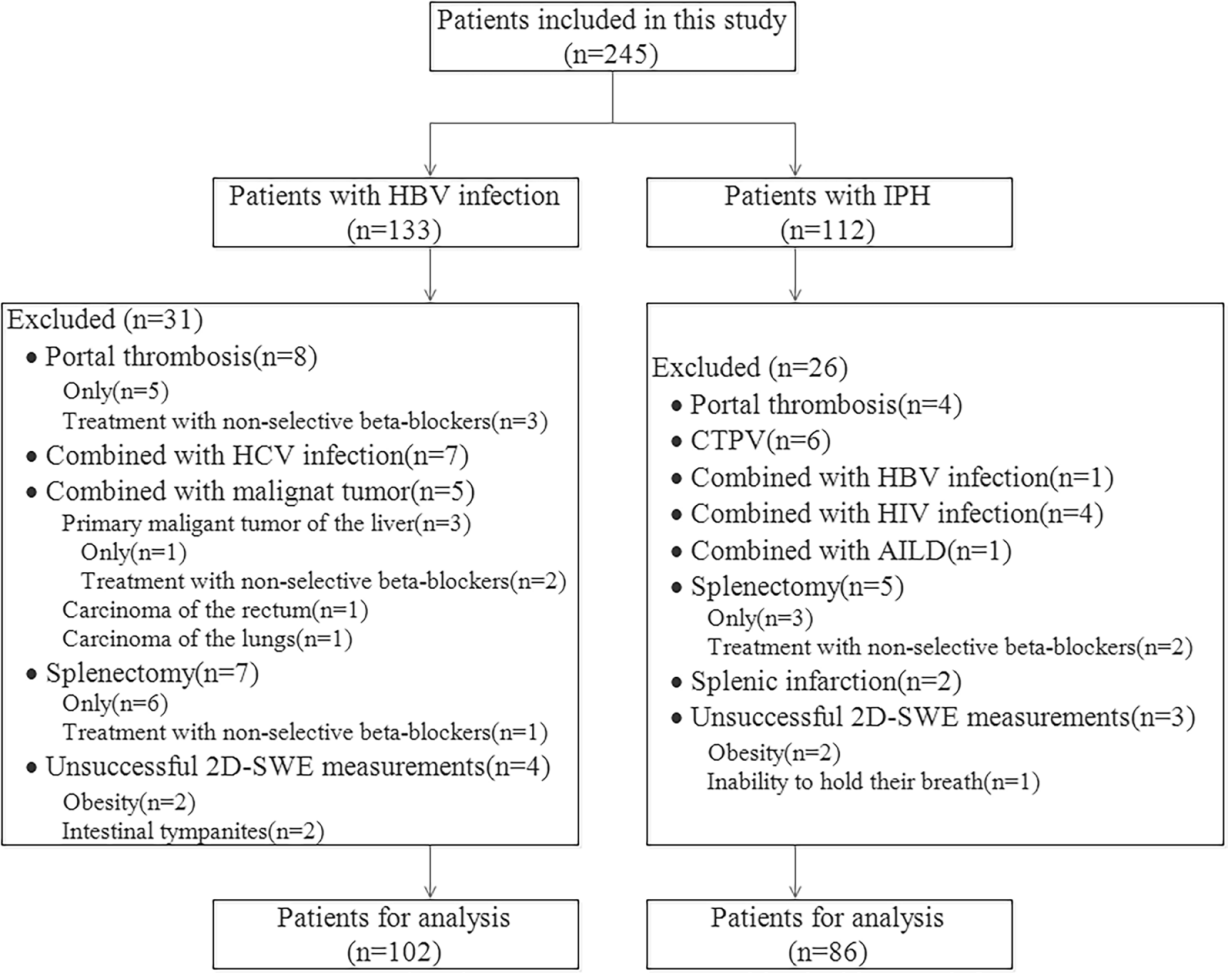
The results of the study patient enrolments. *Note:* HBV, hepatitis B virus; HCV, hepatitis C virus; 2D-SWE, two-dimensional shear wave elastography; IPH, idiopathic portal hypertension; CTPV, cavernous transformation of portal vein; AILD, autoimmune liver disease

Over the study period, up to 245 potentially eligible patients were retrospectively enrolled in our study. Among them, 26 and 31 patients were excluded in the IPH and HBV-infected patients, respectively, because of the portal thrombosis only, portal thrombosis and non-selective beta-blockers, combination with other chronic diseases, malignant tumor, hepatocellular carcinoma and non-selective beta-blockers, splenectomy only, splenectomy and non-selective beta-blockers, and unsuccessful 2D-SWE measurements. Finally, 188 patients were enrolled for the final statistical analysis (Fig. 1), including 86 IPH patients [mean age 47.5 ± 12.2 years; male 45 (52.3%)] and 102 HBV-infected patients [mean age 50.3 ± 11.1 years; male 68 (66.7%)] (Table 1). In IPH patients, 30 patients (34.9%) had HRV. In HBV-infected patients, 47 patients (46.1%) had HRV. Patients’ demographics are shown in Table 1.

**Table 1:** Baseline characters of patients

Variables	HBV (n = 102)	IPH (n = 86)
Age (year)	50.3 ± 11.1	47.5 ± 12.2
No. of male	68 (66.7%)	45 (52.3%)
GEV		
Non-HRV	55 (53.9%)	56 (65.1%)
HRV	47 (46.1%)	30 (34.9%)
2D-SWE (kPa)		
LS	16.2 ± 4.3	9.4 ± 2.4
SS	35.5 ± 9.8	39.3 ± 9.9
Conventional US		
Spleen size (cm)		
Thickness	4.9 ± 1.0	5.0 ± 0.7
Diameters	17.0 ± 4.4	15.9 ± 3.4
PVF (ml/min)	1882.0 (1424.3–2897.0)	2008.0 (1171.0–2898.0)
SVF (ml/min)	1022.0 (626.0–2129.5)	1213.0 (672.0–1763.0)
Laboratory values		
ALT (IU/L)	24.5 (17.8–34.0)	19.0 (13.0–24.0)
ALB(g/L)	35.9 (31.9–40.6)	39.1 (33.5–43.2)
AST(IU/L)	31.0 (22.0–37.5)	23.0 (19.0–28.0)
TBIL (µmol/L)	20.2 (13.6–31.3)	17.7 (13.3–22.2)
DBIL (µmol/L)	10.3 (7.0–15.5)	7.2 (5.7–8.8)
IBIL (µmol/L)	10.2 (6.5–17.1)	10.9 (7.5–14.3)
ALP(IU/L)	80.0 (60.3–102.0)	74.0 (56.0–110.0)
GGT(IU/L)	39.0 (22.0–61.8)	30.0 (22.0–51.0)
PLT (10^9^/µl)	49.0 (32.8–76.8)	62.0 (41.0–81.0)
PT(s)	16.9 (15.8–18.4)	14.8 (12.9–15.2)

Continuous variables were expressed as means ± standard deviations (SD) or medians [interquartile ranges (IQR)], and categorical variables were expressed as n(%), when appropriate

HBV, hepatitis B virus; IPH, idiopathic portal hypertension; GEV, gastroesophageal varices; Non-HRV, without high-risk varices; HRV, high-risk varices; LS, liver stiffness; SS, spleen stiffness; 2D-SWE, two-dimensional shear-wave elastography; PVF, portal venous flow volume; SVF, splenic venous; ALT, alanine aminotransferase; ALB, albumin; AST, aspartate aminotransferase; TBIL, total bilirubin; DBIL, direct bilirubin; IBIL, indirect bilirubin; ALP, alkaline phosphatase; GGT, glutamyl transpeptidase; PLT, platelet count; PT, prothrombin time

### Difference between IPH and HBV-infected patients with and without HRV

**Fig. 2:**
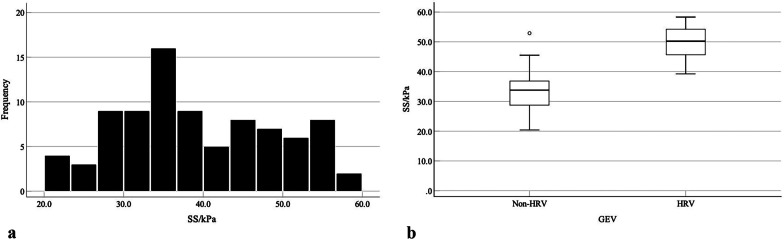
**a** Histogram and **b** Tukey box show the distribution of spleen stiffness values measured by using 2D-SWE in IPH. Dots in b show the individual patient spleen stiffness values, box boundaries show the first and third quartile values, and the whiskers show 1.5 times the interquartile range

**Fig. 3:**
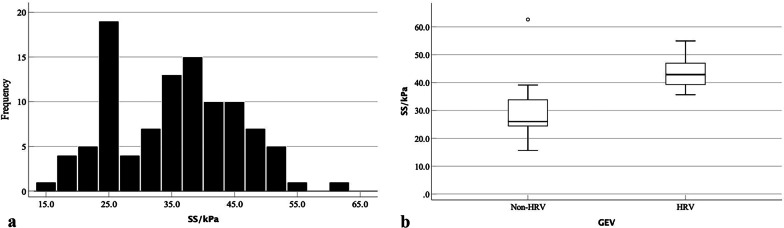
**a** Histogram and **b** Tukey box show the distribution of spleen stiffness values measured by using 2D-SWE in HBV-infected patients. Dots in b show individual patient spleen stiffness values, the box boundaries show the first and third quartile values, and the whiskers show 1.5 times the interquartile range

In patients without HRV, the patients with IPH reveal preserved liver function and blood coagulation function (*p* < 0.0001), larger spleen thickness (5.0 ± 1.0 cm vs. 4.0 ± 1.0 cm, *p* < 0.05), higher values of SS (33.8 kPa vs. 26.0 kPa, *p* < 0.0001), and lower values of LS (8.9 kPa vs. 15.3 kPa, *p* < 0.0001) compared with HBV-infected patients. However, the flow volumes in portal venous system were not statistically difference between the IPH and HBV-infected patients (*p* > 0.05) (Table 2; Figs. 2, 3).

**Table 2:** Variables in IPH and HBV-infected patients without or with high-risk varices

Variables	Non-HRV			HRV		
	HBV(n = 55)	IPH(n = 56)	*P* values	HBV(n = 47)	IPH(n = 30)	*P* values
Age (year)	49.7 ± 10.7	47.9 ± 10.8	0.386	51.1 ± 11.6	46.8 ± 14.6	0.152
No. of male	34 (61.8%)	29 (51.8%)	0.288	34 (72.3%)	16 (53.3%)	0.090
2D-SWE (kPa)						
LS	15.3 (12.5–16.7)	8.9 (6.2–10.1)	< 0.0001	18.3 (14.4–21.6)	11.1 (9.6–12.2)	< 0.0001
SS	26.0 (24.4–34.0)	33.8 (28.5–36.9)	< 0.0001	42.9 (39.2–47.0)	50.2 (45.6–54.3)	< 0.0001
Conventional US						
Spleen size (cm)						
Thickness	4.0 ± 1.0	5.0 ± 1.0	0.012	5.6 ± 0.9	5.5 ± 0.7	0.758
Diameters	14.1 ± 3.1	14.5 ± 2.0	0.452	20.8 ± 2.7	18.5 ± 3.9	0.004
PVF (ml/min)	1371.0 (728.5–2102.5)	1871.0 (1037.5–3460.5)	0.188	1904.0 (1570.5–2983.5)	2115.0 (1207.8–2915.3)	0.757
SVF (ml/min)	966.0 (444.0–2384.5)	833.0 (373.0–1839.5)	0.235	1072.0 (701.5–2086.5)	1224.5 (699.0–1848.8)	0.995
Laboratory values						
ALT (IU/L)	23.0 (11.5–61.0)	20.0 (18.5–31.0)	0.002	25.0 (18.0–32.0)	21.0 (14.5–26.0)	0.067
ALB (g/L)	37.3 (30.0–43.3)	39.1 (36.8–42.6)	0.001	35.0 (31.8–39.8)	39.3 (33.0–44.7)	0.050
AST (IU/L)	28.0 (23.0–46.5)	26.0 (20.0–34.0)	< 0.0001	31.0 (22.0–37.0)	23.0 (19.8–28.0)	0.015
TBIL (µmol/L)	24.1 (16.2–37.3)	20.6 (16.0–23.1)	0.391	20.0 (13.6–30.3)	16.1 (12.7–23.9)	0.222
DBIL (µmol/L)	8.9 (7.7–11.1)	7.8 (6.8–9.6)	< 0.0001	10.3 (7–16.1)	6.5 (5.6–9.3)	0.010
IBIL (µmol/L)	19.1 (8.1–24.3)	11.3 (10.6–14.8)	< 0.0001	10.6 (6.5–16.1)	9.9 (6.6–14.3)	0.620
ALP (IU/L)	75.0 (56.5–102.5)	87.0 (58.5–106.5)	0.438	80.0 (61.0–102.0)	75.0 (57.5–112.0)	0.548
GGT (IU/L)	46.0 (26.5–53.0)	44.0 (22.0–90.0)	0.824	32.0 (19.0–64.0)	30.5 (22.8–53.0)	0.975
PLT (10^9^/µl)	44.0 (25.0–90.5)	81.0 (62.0–87.0)	< 0.0001	53.0 (36.0–75.0)	52.5 (38.8–77.0)	0.967
PT (s)	17.3 (16.3–17.9)	13.4 (12.8–14.9)	< 0.0001	16.7 (15.5–18.8)	15.1 (13.5–15.5)	< 0.0001

Continuous variables were expressed as means ± standard deviations (SD) or medians [interquartile ranges (IQR)], and categorical variables were expressed as n(%), when appropriate

HBV, hepatitis B virus; IPH, idiopathic portal hypertension; GEV, gastroesophageal varices; Non-HRV, without high-risk varices; HRV, high-risk varices; LS, liver stiffness; SS, spleen stiffness; 2D-SWE, two-dimensional shear-wave elastography; PVF, portal venous flow volume; SVF, splenic venous; ALT, alanine aminotransferase; ALB, albumin; AST, aspartate aminotransferase; TBIL, total bilirubin; DBIL, direct bilirubin; IBIL, indirect bilirubin; ALP, alkaline phosphatase; GGT, glutamyl transpeptidase; PLT, platelet count; PT, prothrombin time

In patients with HRV, the patients with IPH had significantly lower values of LS (11.1 kPa vs. 18.3 kPa, *p* < 0.0001), higher values of SS (50.2 kPa vs. 42.9 kPa, *p* < 0.0001), smaller spleen diameters (18.5 ± 3.9 cm vs. 20.8 ± 2.7 cm, *p* < 0.05), and shorter PT (15.1 s vs. 16.7 s, *p* < 0.0001). No statistically difference were found regarding the liver function, platelet counts, spleen thickness, and flow volumes in portal venous system (p > 0.05) (Table 2; Figs. 2, 3).

### Evaluating the diagnostic performance of SS in comparison with LS, SS/LS, LSPS, PH risk score and varices risk score in IPH and HBV-infected patients

**Fig. 4:**
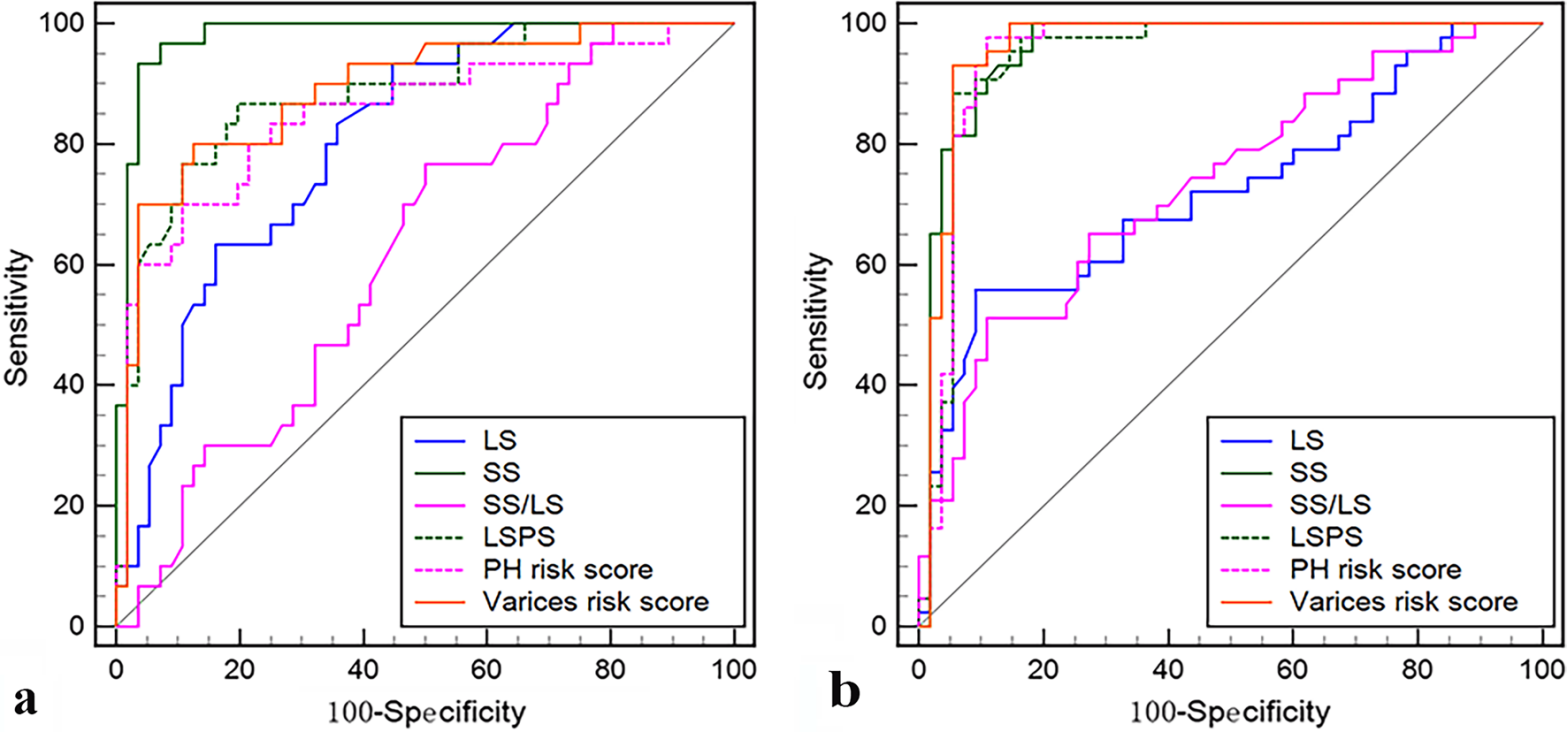
Area under the receiver operating characteristic curves for the prediction of the presence of HRV in IPH (**a**) and HBV-infected patients (**b**). *Note:* LS, liver stiffness; SS, spleen stiffness; SS/LS, spleen stiffness-to-liver stiffness ratio; PH risk score, portal hypertension risk score; LSPS, liver stiffness spleen-diameter-to-platelet-ratio score

In the IPH patients, SS demonstrated the highest diagnostic performance compared with the other noninvasive parameters for predicting the presence of HRV, and the differences in the AUCs were all statistically significant (Table 3; Fig. 4). The AUC of SS was 0.98 for predicting the presence of HRV, with 0.93 sensitivity, 0.96 specificity, 0.93 PPV and 0.96 NPV, and the best cutoff value was 44.0 kPa (Table 3).

**Table 3:** Performance of noninvasive parameters for the prediction of high-risk varices

Variables	n(P)	Cutoff value	AUC	Sensitivity	Specificity	PPV	NPV	LR+	LR−
SS									
IPH	30 (34.88)	44.0	0.98 (0.92–1.00)	0.93 (0.78–0.99)	0.96 (0.88–1.00)	0.93 (0.78–0.98)	0.96 (0.88–0.99)	0.26 (0.07–1.02)	0.00 (0.00–0.00)
HBV	47 (46.1)	35.2	0.96 (0.90–0.99)	1.00 (0.93–1.00)	0.82 (0.69–0.91)	0.82 (0.72–0.89)	0.98 (0.87–1.00)	0.06 (0.03–0.10)	0.00 (0.00–0.01)
LS									
IPH	30 (34.88)	9.1	0.81*** (0.71–0.88)	0.93 (0.78–0.99)	0.55 (0.42–0.69)	0.53 (0.45–0.60)	0.94 (0.80–0.98)	0.02 (0.02–0.03)	0.00 (0.00–0.01)
HBV	47 (46.1)	17.7	0.72*** (0.62–0.81)	0.57 (0.42–0.71)	0.91 (0.80–0.97)	0.84 (0.69–0.93)	0.71 (0.64–0.78)	0.06 (0.03–0.15)	0.47 (0.00–0.01)
SS/LS									
IPH	30 (34.88)	4.1	0.62*** (0.51–0.73)	0.77 (0.58–0.90)	0.50 (0.36–0.64)	0.45 (0.37–0.53)	0.80 (0.67–0.89)	0.02 (0.01–0.02)	0.01 (0.00–0.01)
HBV	47 (46.08)	2.5	0.73*** (0.64–0.82)	0.49 (0.34–0.64)	0.89 (0.78–0.96)	0.79 (0.63–0.90)	0.67 (0.60–0.73)	0.04 (0.02–0.10)	0.01 (0.00–0.01)
LSPS									
IPH	30 (34.88)	2.3	0.88* (0.80–0.94)	0.87 (0.69–0.96)	0.80 (0.68–0.90)	0.79 (0.64–0.89)	0.88 (0.79–0.93)	0.04 (0.03–0.08)	0.00 (0.00–0.00)
HBV	43 (43.88)	3.9	0.94 (0.88–0.98)	0.88 (0.75–0.96)	0.95 (0.85–0.99)	0.93 (0.81–0.98)	0.91 (0.82–0.96)	0.16 (0.05–0.49)	0.00 (0.00–0.00)
PH risk score									
IPH	30 (34.88)	4.4	0.85** (0.76–0.92)	0.70 (0.51–0.85)	0.89 (0.78–0.96)	0.78 (0.61–0.89)	0.85 (0.76–0.91)	0.06 (0.03–0.14)	0.00 (0.00–0.01)
HBV	43 (43.88)	3.8	0.95 (0.88–0.98)	0.98 (0.88–0.99)	0.89 (0.78–0.96)	0.88 (0.77–0.94)	0.98 (0.88–1.00)	0.09 (0.04–0.19)	0.00 (0.00–0.00)
Varices risk score									
IPH	30 (34.88)	0.9	0.89* (0.80–0.95)	0.80 (0.61–0.92)	0.88 (0.76–0.95)	0.77 (0.63–0.88)	0.89 (0.80–0.94)	0.06 (0.03–0.13)	0.00 (0.00–0.00)
HBV	43 (43.88)	1.7	0.96 (0.90–0.99)	0.93 (0.81–0.99)	0.95 (0.85–0.99)	0.93 (0.82–0.98)	0.95 (0.85–0.98)	0.17 (0.06–0.51)	0.00 (0.00–0.00)

Statistical quantifications were demonstrated with 95% CI, when applicable. AUC of SS was statistically compared with AUC of LS, SS/LS, LSPS, PH risk score and varices risk score, respectively

SS, spleen stiffness; LS, liver stiffness; SS/LS, spleen stiffness-to-liver stiffness ratio; LSPS, LS spleen-diameter-to-platelet-ratio score; PH risk score, portal hypertension risk score; n, number of patients; P, prevalence; AUC, areas under the receiver operating characteristic curve; PPV, positive predictive value; NPV, negative predictive value; LR+, positive diagnostic likelihood ratio; LR−, negative diagnostic likelihood ratio

*P < 0.05; **P < 0.001; ***P < 0.0001

In the HBV-infected patients, compared with LS, SS/LS, LSPS, PH risk score and varices risk score, the AUC of SS reached 0.96 for predicting the presence of HRV, with 1.00 sensitivity, 0.82 specificity, 0.82 PPV and 0.98 NPV, and the best cutoff value was 35.2 kPa (Table 3). SS still demonstrated the highest AUC and was significantly higher than LS and SS/LS, whereas no significant difference was observed between AUCs of SS and LSPS, PH risk score and varices risk score (Table 3; Fig. 4).

## Discussion

This retrospective single-center study focused on patients with IPH by using 2D-SWE to predict the presence of HRV, as compared to HBV-infected patients. Accurate evaluation of the presence of HRV is of great importance. Herein, for the first time, we analyzed the diagnostic performance of SS by using 2D-SWE in patients with IPH compared to those with HBV.

For evaluating the presence of HRV, SS showed the highest performance compared with other noninvasive parameters (AUC: 0.98) in IPH. Virginia Hernándea-Gea et al. regarded the natural history of patients with non-cirrhotic portal hypertension and found that the SS was markedly increased in the early stages of the disease [15], this finding could support our conclusion. Additionally, these pathophysiological studies have been demonstrated by the findings of several studies. At the early stage of IPH, the gross histological features of the liver are associated with the intrahepatic vascular alterations, which belong to Glisson’s sheath. The intrahepatic vein branches present sclerotic, vein wall thicken, obliteration, and early slight lymphoid cell infiltration of the portal tracts and branches [16]; furthermore, the liver function tends to be typically preserved or slightly deranged [1]. Our results regarding liver function are consistent with those previously reported (Table 2). The above natural history leads to the main clinical features that are associated with PH at the early stage of IPH, including splenomegaly, hypersplenism, and variceal vein [1]. Therefore, the SS is noticeably changed in patients with IPH.

In HBV-infected patients, the SS was the best potential noninvasive parameter for evaluating the presence of HRV (AUC 0.96). On the one hand, SS provided the highest AUC compared with LS and SS/LS; on the other hand, although no significant difference was observed between the AUCs of SS, LSPS, PH risk score and varices risk score, the SS by using 2D-SWE could be more easily performed in clinical compared with other parameters. When patients are in the early stage of HBV-infections, the hallmarks of liver are mostly present in the hepatocytes, as opposed to the portal tracts in IPH [17, 18]. Additionally, the volume of hepatocytes accounts for more than 90% of the total volume of the liver, with liver function being more severely affected in HBV-infected patients than in those with IPH. In the liver, there is mostly inflammation, thick fibrous septa, and small nodules, which are the most important factors for the LS increase [19], as found in our study (Table 2). However, at the later stages, with the progression of hepatocytes death, extracellular matrix deposition, and vascular reorganization, the pathological hallmark of the liver is pseudolobule formation, which includes regenerative nodules, fibrous septa, and microvascular clotting [16]. Finally, the irreversible histological aberrations mentioned above drive the increased intrahepatic resistance to the onset of complications of portal hypertension. As discussed above, increased portosystemic collaterals flow and the complications will appear, including upper gastrointestinal variceal bleeding, splenomegaly, hypersplenism, portosystemic collaterals and ascites (Table 2). With the progression of portal pressure, the severity of portal pressure partially depends on extrahepatic elements that are closely related to blood flow, including splanchnic vasodilatation, hyperdynamic circulation, and portosystemic collaterals [20]. Finally, the correlation between LS and portal hypertension may be lost. Kumar and Reiberger et al. showed that the relationship between LS and portal pressure will be lost with increased portal pressure in cirrhosis [19–22]. In a study of HCV-infected patients, the researcher found that there was a correlation between LS and the presence of GEV, but no relationship between LS and the GEV’s size was observed [23]. The above conclusions agree with the findings from this study.

The study has several limitations. Firstly, the sample size was small because IPH is a rare disease. Secondly, this study is a retrospective single-center study. As described above, these limitations may limit the representativeness of the conclusions. However, our threshold has a strict quality control: (1) the UGE examinations were performed by two experienced specialists; (2) the US and 2D-SWE examinations of all patients were conducted by two experienced sonographers. The above characteristics contributed to avoid inter observer variation. Lastly, the study was a retrospective analysis. Hence, further well-designed prospective multicenter study will be needed to verify the conclusions in this study.

## Conclusion

In conclusion, our study demonstrated that SS using 2D-SWE shows the best diagnostic performance for predicting the presence of HRV in IPH and HBV-infected patients.

## Data Availability

The data generated and/or analyzed during the current study are available from the corresponding author on reasonable request.

## Abbreviations

ARFI: Acoustic radiation force impulse imaging
AUCs: Areas under the receiver operating characteristic curves
CT: Computed tomographic
2D-SWE: Two-dimensional shear-wave elastography
GEV: Gastroesophageal varices
HRV: High-risk varices
HE: Hepatic encephalopathy
HBV: Hepatitis B virus
IPH: Idiopathic portal hypertension
LS: Liver stiffness
LSPS: LS spleen-diameter-to-platelet-ratio score
PH: Portal hypertension
pSWE: Point shear wave elastography
ROC: Receiver operating characteristic curves
ROI: Region of interest
SS: Spleen stiffness
TE: Transient elastography
UGE: Upper gastrointestinal endoscopy
